# Cancrum Oris

**Published:** 1848-10

**Authors:** 


*Cancrum Oris.*—"Mr. Micklethwait detailed the case of a child, aged five, who suffered from hooping cough and tubular bronchitis, to whom he gave, in the space of ten days, a drachm of mercury with chalk. The mouth became sore, without any flow of saliva. The soreness became confined to the left cheek, which soon presented the appearance of cancrum oris, which destroyed the cheek, opened the commissure of the lips, and destroyed the gums, teeth, and part of the tongue of that side, and terminated fatally in five days after the appearance of the gangrene. The child complained of excruciating pains of the hands and feet, which directly after death became very black. It had suffered from erysipelas of the same cheek three years ago, and ever since, that side had been less in size than the other."—*Medical Times.*

